# Peer-based Promotion and Nurse-led Distribution of HIV Self-Testing Among Networks of Men in Dar es Salaam, Tanzania: Development and Feasibility Results of the STEP Intervention

**DOI:** 10.21203/rs.3.rs-3283552/v1

**Published:** 2023-08-22

**Authors:** Donaldson F. Conserve, Gamji Rabiu Abu-Ba’are, Samuel Janson, Frank Mhando, Grace V. Munisi, Busara Drezgic, Abubakar Rehani, Wynton Sims, Tiarney Ritchwood, Augustine Choko, Stella Mushy, Cheryl Johnson, Larissa Jennings Mayo-Wilson, Albert Komba, Peris Urasa, LaRon Nelson, Gaspar Mbita

**Affiliations:** The George Washington University; Rochester University; The George Washington University; University of Johannesburg; EngenderHealth -Tanzania; EngenderHealth -Tanzania; T-MARC -Tanzania; University of California San Francisco; Duke University; Malawi-Liverpool Wellcome Trust Clinical Research Programme; Muhimbili University of Health and Allied Sciences; World Health Organization; University of North Carolina at Chapel Hill; Jhpiego Tanzania - An Affiliate of Johns Hopkins University; Ministry of Health; Yale University School of Nursing; Vrije Universiteit

**Keywords:** HIV, Men, HIV self-testing, Tanzania

## Abstract

**Background:**

According to the 2016–2017 Tanzania HIV Impact Survey, only 45% of men living with HIV (MLWH) were aware of their HIV status. In an effort to increase HIV testing in Tanzania, including among men, the Government of Tanzania passed a law in December 2019 to allowing HIV self-testing (HIVST) to be included in the national testing strategies. The objective of this paper is to describe the development and pilot feasibility assessment of the Self-Testing Education and Promotion (STEP) intervention, which was one of the projects conducted in Tanzania focusing on men to inform policy change.

**Methods:**

The development and piloting processes were guided by the ADAPT-ITT model and informed by a national PEPFAR/USAID-funded HIV implementation science project called *Sauti*.

The adapted STEP intervention included the following two components: 1) peer-based HIVST promotion; and 2) nurse-led HIVST distribution. For the feasibility assessment, 25 men were selected and trained to promote HIVST among their peers before helping to recruit 253 men to receive instructions and collect an HIVST kit from a nurse at a community-based study tent site.

**Results:**

Of the 236 participants who completed the 1-month follow-up survey, 98.3% reported using the kit. The majority (92.4%) of participants reported a negative HIVST result while 4.2% (n=10) received a positive result. Most (70%, n=7) of the participants with a positive result sought follow-up services at a healthcare facility while 40.3% (n=95) of the participants with a negative self-test result visited the community-based project site. Most of the men (53%, n =129) did not visit a healthcare facility or the study site. The majority of participants reported having a mobile phone and forty-seven of them called someone to share their results while twenty-seven sent a text message about their results.

**Conclusion:**

The findings demonstrate that the combined peer-based promotion and nurse-led distribution of HIVST intervention in the community for men was acceptable and feasible. However, the high proportion of men who visited the tent site in the community after self-testing indicated that future research should evaluate the potential for nurses to provide community-based linkage to HIV care and prevention services for self-testers.

## Introduction

The 2016–2017 Tanzania HIV Impact Survey found that only 52.2% of people living with HIV (PLWH) ages 15 to 64 years knew their status (1). Men were less likely to know their HIV status compared to women (46% versus 57%) and less likely, upon knowing their status, to initiate antiretroviral therapy (ART) (86% versus 92%) (1). Consequently, there was a need for new strategies designed to improve case-finding, especially among men, and move towards epidemic control. To address this need, the Government of Tanzania (GoT) and Tanzania Commission of AIDS (TACAIDS) collaborated with Family Health International (FHI) 360 to develop, implement, and scale up *Furaha Yangu! (My Happiness!)*, a national campaign to promote HIV Test and Treat services with a focus on reaching men (2, 3). Additionally, the GoT supported the development of the 2020 Male Catch-Up Plan, which provided national strategies to reach heterosexual men with HIV services (4).

Although these efforts have been successful in increasing HIV testing services in Tanzania among men, HIV self-testing (HIVST) was not included due to a lack of policy in the country for its rollout, and a dearth of evidence supporting the feasibility and acceptability within the population (5). Global evidence had shown that HIVST, which allows individuals to test for HIV in privacy, is acceptable, feasible, and effective in increasing HIV testing (6–8). However, the lack of country-specific evidence for HIVST contributed to a delay in introducing and integrating HIVST into the national policy in Tanzania. Prior to approval of HIVST in Tanzania, the 2008 National HIV Act required that HIV testing be conducted by a healthcare professional. In order to generate country-specific data on different factors relating to HIVST implementation, Jhpiego Tanzania collaborated closely with the National AIDS Control Program (NACP) to conduct a national HIVST demonstration project as a part of *Sauti* (meaning “voice of the people”).

The *Sauti* Project was launched in 2014 with funding from the President’s Emergency Plan for AIDS Relief and U.S. Agency for International Development (PEPFAR/USAID) (9). *Sauti* was a community-based HIV combination prevention project, which offered clinical and structural support services to key and vulnerable populations (KVPs) in 14 regions (9). The *Sauti* Project was also involved in implementing the PEPFAR’s Dreams Initiative (Determined, Resilient, Empowered, AIDS-free, Mentored, and Safe), which focused on adolescent girl and young women (AGYW) and offered comprehensive, evidence-based HIV prevention and treatment packages (10) Services included HIV prevention, testing, care, and treatment, and were offered to *Sauti* beneficiaries through mobile outreach units working in hot spots such as truck stops, brothels, bars, mining centres, nightclubs, guest houses, and truck drivers’ parking places (9). The services were designed to be stigma-free by utilizing health care providers trained to work with populations that experience barriers to accessing reproductive health services (9). To ensure improved linkage to care, *Sauti* Project also offered transportation support or accompaniment by peer educators and home-based care providers (HBCs) for all female sex workers (FSWs) newly diagnosed with HIV infection to nearby health facilities for follow-up care.

Building on these services and evidence from other countries, *Sauti* Project advocated in 2017 for the inclusion of HIV self-testing (HIVST) in the HIV prevention portfolio in order to expand access to HIV testing services (HTS) among KVPs who may experience barriers to accessing health facilities or community-based health services. In March 2018, the *Sauti* Project team introduced HIVST in Dar es Salaam, Iringa, Morogoro, Njombe, and Tabora where the project was also offering comprehensive combination prevention services. Lessons learned from the initial national demonstration of HIVST allowed expansion to eight other regions. Distribution models were primarily community-based with trained nurses and community-based HIV service providers (CBHSPs formerly referred to as peer educators) delivering HIVST kits in the community and through HIV prevention peer education sessions. Clinical HIV testing providers working in the community also integrated HIVST with conventional HTS by offering HIVST kits to newly diagnosed individuals for secondary distribution to their peers or partners. A total of 35,137 clients received HIVST kits across 13 regions. However, given the focus of the *Sauti* Project to provide services for FSWs, MSM, and AGYW, heterosexual men were not included as a priority population for the national HIVST demonstration project. Therefore, there was a lack of country-specific evidence for how best to reach heterosexual men for primary distribution of HIVST kits.

In order to address the lack of data on the feasibility of distributing HIVST among heterosexual men, this study, informed by the ADAPT-ITT model, aimed to develop and pilot a community-based HIVST intervention for heterosexual men. The ADAPT-ITT model utilizes an eight-phase process that can be used in whole or parts to adapt evidence-based interventions for a population and context of interest (11). The 8 phases include the following: 1) Assessment; 2) Decision; 3) Adaptation; 4) Production; 5) Topical experts; 6) Integration; 7) Training; and 8) Testing (11). The intervention is called **S**elf-**T**esting **E**ducation and **P**romotion (STEP), locally referred to as *Mate Yako Afya Yako* (5). The objective of this paper is to describe the use of the ADAPT-ITT model to guide the development and pilot testing of the intervention for feasibility among social networks of men in Dar es Salaam, Tanzania.

## Methods

### Setting

The setting for this study was the Kinondoni Municipality of Dar es Salaam, Tanzania. Participants were recruited from “camps” located in this area. Camps are social groups consisting primarily of young men who often meet daily and are governed by leaders elected from within the camp (12). The research team chose to engage camps for the recruitment and training of CBHSPs and intervention participants due to the input of team members who had familiarity with these social networks, as well as research indicating that camps can serve as strategic venues for promoting HIV testing and education (13). The camps included in this study were identified through cluster randomized cluster trial (cRCT) assessing the efficacy of a combined microfinance and HIV prevention intervention (14). The contact information collected through the cRCT was used for recruitment in the STEP intervention. Participants from the camps were first recruited into the formative study (Assessment Phase) (5), and later into the intervention following the recruitment and training of CBHSPs.

### Phases of ADAPT-ITT model for STEP Intervention

Completion of the eight phases of the ADAPT-ITT model for the STEP intervention occurred over 4 years (2015–2019). Phase I (Assessment) involved conducting a cross-sectional survey and in-depth interviews with men in between 2015 and 2017 to assess their perceptions of HIVST, willingness to self-test (15), and their recommendations for the intervention (5). Phase II (Decision) involved meeting with key stakeholders, including from the National AIDS Control Program (NACP), in 2018 to decide on the key components for the intervention. Phase III (Adaptation) included the adaptation of *Sauti*’s community-based HIV prevention intervention for the STEP intervention. The community engagement strategies for the *Sauti* Project were informed by the *Engaging Men at the Community Level* training manual created by EngenderHealth and Promundo (16). In addition, the *Sauti* Project was using the “*National Training for HIV Prevention using HIV Self Testing*” manual developed by the National AIDS Control Program (NACP) as part of the national HIVST demonstration project.

We reviewed the *Engaging Men at the Community Level* and *National Training for HIV Prevention using HIV Self Testing* manuals and made the necessary adaptations for the STEP intervention training modules. Table 1 describes the old modules and the adaptations made for the STEP intervention modules. As shown in Table 1, two new modules, Module V and Module VI, were added as part of the adaptation to teach participants effective communication and counseling skills and provide information to male CBHSPs on the definition of HIVST, the benefits, and the different types of self-testing methods, along with an HIVST demonstration using the OraQuick Rapid HIV-1/2 Antibody Test, OraSure Technologies. This demonstration includes using the test and interpreting the results. Next, participants receive information about the benefits of engaging in HIV care and initiating treatment. In addition, the team decided to include a nurse-based HIVST distribution component of the STEP intervention to align with the national HIVST demonstration project being implemented as part of *Sauti* project.

#### Description of adapted STEP Intervention.

Phase IV (Production), which also occurred in 2018, entailed production of the adapted intervention manual. The adapted intervention included two components. The first component, which is the peer-based HIVST promotion, consists of selecting and training CHBSPs to promote HIVST using the training modules. After training, CHBSPs promote HIVST among their social network members and create demand before helping to recruit network members to enroll in the study and receive HIVST kits from a study team member and HIV counselor. The second component of the intervention, which was also informed by the national HIVST demonstration project, includes nurse-led HIVST distribution to participants. A nurse trained on HIVST works closely with the CHBSPs to carry out the distribution of the HIVST kits at a tent located in the community near where participants live. Before providing the HIVST kit to the participant, the nurse demonstrates how to use the HIVST kit with a sample HIVST kit and confirms that the participants understand the instructions. In addition, the nurse informs the participants to seek follow-up services such as confirmatory testing and linkage to care, if needed, at nearby health facilities that provide HIV testing and treatment and the need to test again in three months since HIVST does not detect acute HIV infection.

In Phases V and VI (Topical experts and Integration), the research team collected feedback from topical experts and community stakeholders pertaining to the adapted STEP intervention manual. Phases VII and VIII (Training and Pilot Testing) occurred in 2019 and involved the selection and training of 25 CBHSPs within their camp communities (curriculum, training agenda, and recruitment criteria are detailed in Tables 1–3). During these phases, CBHSPs engaged in demand generation for HIVST through education and social influence strategies, and eventual recruitment of participants to participate in the nurse-led HIVST distribution component. Table 4 further details the project activities related to each of the phases of the ADAPT-ITT model as they relate to the STEP intervention.

### Inclusion of Participants (Phase VIII)

In June 2019, participants were recruited with the assistance of CHBSPs who referred potential participants to the study team. Based on a recommendation from our formative research that participants should be screened for suicidal ideation to prevent self-harm in case of a positive self-test result at home, camp members were screened for suicidal ideation. Individuals were excluded if they did not meet all inclusion criteria; were unable to participate due to psychological disturbance, cognitive impairment, or threatening behavior; or reported positive HIV status. Written informed consent was obtained from all participants.

### Data collection and analysis (Phase VIII)

#### Baseline Survey

The baseline survey was conducted during the month of June 2019 and covered demographic profile, HIV-related risk behaviors, HIV testing history, reasons for not having tested, and self-perceived HIV risk. Each interview lasted 35 to 45 minutes. At baseline, the names (or nicknames) and contact information (mobile number) of the participants were collected to ensure successful follow-up with participants for the one-month survey.

#### One-month follow-up survey

A follow-up interview was conducted in August 2019 to determine whether participants had used the HIVST kits provided and their experience with it, their self-reported test result, potential harms, confirmatory testing (if positive), linkage to ART, and willingness to pay. The questionnaires were administered by a research assistant in Kiswahili language on Qualtrics via a Samsung Tablet. All participants provided written informed consent and were reimbursed approximately 10,000 Tanzanian Shillings/USD 4.28 for each of the baseline and follow-up surveys. Data were analyzed using STATA software version 15.0 (Stata Statistical Software: Release 15. 2017. College Station, TX: StataCorp LLC.) Following data cleaning and checking for consistency and completeness, descriptive statistics were summarized and frequency and percentages were used to describe the characteristics of the study population and their perceptions and behaviors related to self-testing.

## Results

In total, 252 men received HIVST kits and 236 men were reached for the one-month follow-up. Men who did not report for the one-month follow-up were excluded from the baseline and follow-up survey data (Table 5). Of the 236 men in the final sample, over half (54.3%) were under the age of 28. Similarly, over half (52.1%, n = 123) of the participants had either completed primary school or received less education. Most of participants (60.2%, n = 142) had never been married and over half (56.8%, n = 134) had a paying job. The majority of the participants surveyed (83.9%, n = 198) had sexual partners. Approximately half (52.5%, n = 124) of men reported never using condoms, while 18.2% (n = 43) always use condoms during sexual intercourse. Many of the men 46.6% (n = 110) reported that they knew their partner’s HIV status. More than half (53.8%, n = 127) of the participants reported having heard of HIVST and the majority (84%, n = 200) reported being encouraged by a peer to take an HIVST kit.

Table 6 shows the self-reported behavior of HIVST among participants at 1-month follow-up. Most of them (93.2%, n = 220) used the self-test kit at a private space away from the tent. Only 5.1% (n = 12) used the HIVST kit at the tent and 1.7% (n = 4) reported that they did not use the self-test kit. Majority of the participants 92.4% (n = 218) received a negative result while 4.2% (n = 10) received a positive result. Approximately half of participants 53.0% (n = 125) reported that they did nothing after they received the results. Only 3.0% (n = 7) went to the health facility while 40.3% (n = 95) visited the study tent after receiving their results. Regarding the length of time participants took to use the self-test kit, 2/3rd 67.8% (n = 160) reported that they used the HIVST kit on the same day they received it and 30.5% (n = 72) used it after the first day.

As shown in Table 7, very few participants 1.7% (n = 4) reported that it was difficult to understand the HIVST kit instructions whereas the majority 45.3% (n = 107) reported that it was normal. At least one-quarter (27.1%, n = 64) reported that it was easy to understand the kit’s instructions. Overall, most of the participants (93.2%, n = 220) reported that what they liked about using an HIVST kit was the confidentiality it offered. Approximately, half of the participants (52.5%, n = 124) were very satisfied with the HIVST kit and the majority (87.7%, n = 207) reported that they would like to use an HIVST kit in the future instead of the conventional HIV test and nearly all (92.4%, n = 218) of them would be willing to pay for an HIVST kit.

As shown in Table 8, regarding mobile health and HIVST, most respondents (95.3%, n = 225) reported that they had mobile phones but the majority (91.5%, n = 216) did not use their phones to take pictures of their HIVST results. Of the 9 participants who reported taking a picture of their results, most (77%, n = 7) of them showed the picture of the results to someone. However, only 20% (n = 47) reported that they called someone to share the results and 11% (n = 27) sent text messages to share their HIVST kit results.

## Discussion

The aim of this paper was to describe the use of the ADAPT-ITT model to inform the development and feasibility assessment of the STEP intervention for social networks of men in Dar es Salaam, Tanzania. There were several advantages to using the ADAPT-ITT model in this study. First, the phases of the of the ADAPT-ITT model which includes the engagement of key stakeholders enhanced the likelihood of the intervention being informed by the potential beneficiaries and the national stakeholders who have implemented community-based HIV prevention programs in Tanzania. Second, the ADAPT-ITT model encouraged researchers to consider the relevant theoretical and empirical information prior to embarking on the design and thereafter the integration into the eight-phase process in a systematic manner. Hence, the ADAPT-ITT model was useful for the context-specific needs of the target population for the promotion and use of HIVST. Additionally, the model was critical in garnering precautions offered by the national stakeholders since HIVST was not yet included in the national HIV guidelines.

While previous studies have reported on the HIVST acceptability among men (17, 18), this is one of the first studies to develop a peer-led promotion and nurse-based distribution of HIVST intervention for men in Tanzania with their inputs and guidance from national stakeholders involved in national HIV testing and HIVST programs. This study provides additional evidence for collaboration between researchers and program implementers and demonstrates the acceptability and feasibility of an adapted peer-led promotion and nurse-based distribution of HIVST intervention among social networks of men. The decision to collaborate with national stakeholders and adapt existing community-based HIV and HIVST materials for the STEP intervention was to ensure that the national stakeholders who can use the findings to inform policy change and dissemination and implementation science efforts for HIVST were involved from the beginning as recommended by researchers and policy makers in Tanzania (19).

The adapted STEP intervention also included the goals of the ADAPT-ITT model, which includes suitability of an intervention for adaptation, modification to fit the local cultural settings, and maintaining successful recruitment and retention rates. These objectives were achieved based on the success of male CBHSPs in promotion of HIVST awareness among their peers and assistance with recruitment and follow-up of in their social networks for the intervention. This approach aligned well with the practice of engaging in formal and informal conversations, as well as continuing the practice of accompaniment to the clinic for HIV testing (13). It also builds on the success of prior recruitment of men from similar social networks as community health leaders in a prior intervention (20). Similar social network-based strategies for HIVST promotion and distribution have been reported with success in other parts of Tanzania and other countries (21–24). The Ministry of Health in Tanzania, in collaboration with implementing partners such as FHI 360, has also adopted a social and sexual network-based approach during the scale up of HIV self-testing, which was also shown to be acceptable from the formative baseline research phases of the STEP project (15, 25).

### Limitations and future linkage to HIV care research

The study has several strengths and limitations worth highlighting. The first strength of the study includes the combination of quantitative and qualitative data from the formative research and pilot of the intervention. Another strength is the use of the ADAPT-ITT framework to inform the adaptations of the existing intervention and program manuals for the context of the social networks of men we engaged for the project. Third, the study was also developed and implemented in collaboration with national as well as community-based stakeholders, including Ministry of Health and CSO representatives, HIV program implementing partners, and male CHBSPs. The limitations for the study include that the outcomes of interest such as HIVST use and follow-up behaviors after obtaining HIVST results were self-reported. Thus, there may be an overstated report of HIVST use and lower reporting of HIV positive results. In addition, there was a lack of follow-up confirmatory blood-based testing and objective assessment of linkage to care for the participants who reported a positive self-test result. However, this limitation will be addressed a proposed study that will leverage an existing program in Tanzania called nurse-initiated management of ART (NIMART), which has also been implemented in South Africa for over a decade (26–28). The NIMART program can be expanded to allow nurses to provide follow-up services for self-testers at home or any other convenient, safe, and private location selected by a client. A prior study conducted in Tanzania has shown that it is feasible to for nurses to provide community-based antiretroviral therapy (cbART) initiation and that cbART services are preferable than facility-based ART services (29).

Therefore, the proposed study will create the STEP + cbNIMART intervention, which will allow nurses to provide self-testers community-based follow-up services and linkage to care or prevention services. There is evidence that nurses who provide health promotion activities in the field (versus in the clinic) yield superior clinical outcomes than field-based paraprofessional community health workers (30, 31). In addition, evidence from Malawi suggests that when nurses provide home-based follow-up services for self-testers who receive a positive self-test result, they are more likely to start on ART than those who are instructed to seek follow-up services at the facility (32). The STEP + cbNIMART project aims to leverage the evidence from the cbART intervention (29) and the NIMART program to help address the gap for the follow-up services. While registered nurses’ per-hour labor cost are generally higher than paraprofessional community health workers, nurses have been shown to produce overall cost savings through increased efficiency (e.g., effectively handling more cases in less time, addressing complex clinical questions in real-time to support’s patient informed decision-making), higher program retention of patients, and behavioral outcomes that patients self-sustain over longer periods of time (30, 33, 34). Moreover, nurses’ involvement in the STEP + cbNIMART project has the potential to enhance continuity of care following HIVST by serving as a direct conduit to ART for men whose positive self-test results are confirmed, as well as pre-exposure prophylaxis (PrEP) for men whose self-test results are confirmed to be negative but are at high risk for HIV acquisition through factors such as being uncircumcised, having sexually transmitted infection symptoms, and engaging in harmful drinking of alcohol before sex (35). Future studies that investigate potential diminishing economic returns of nurses can also provide important evidence to inform HIV policy, clinical practice protocols, and community-based linkage to ART or PrEP implementation.

## Conclusion

The pilot study has shown that it is acceptable and feasible for a combined peer-led promotion and nurse-based distribution of HIVST intervention for unsupervised use among networks of men in Dar es Salaam, Tanzania. Strategies for reaching men continue to be a priority and social network approaches should be scaled up. Stakeholder-led development, including the engagement of social networks of men and HIV implementing partners, and implementation as informed by the ADAPT-ITT model were important for successful dissemination and countries and programmes should consider this to maximize effectiveness of HIVST programming. Since the completion of the STEP intervention and the PEPFAR-funded national HIVST demonstration project implemented through the *Sauti* project, the National Act was changed in December 2019 to allow HIVST to be offered and Tanzania is a leader in HIVST implementation and scale-up with the goal of increase HIV testing and reducing HIV infections at the population level. The national programme can continue to apply these findings to strategically expand HIVST to close gap in reaching men which remains a priority.

## Figures and Tables

**Table 1:** Description of the Adapted Training Modules for Component I of the STEP Intervention

***Module I: Introductory Activities.* (a) Explores attitudes about gender differences, roles, and inequalities; (b) Understand the difference between “sex” and “gender;” (c) Understand the terms “gender equity” and gender “equality;” (d) Identify the different gender roles and expectations; (e) Understand how gender roles affect the lives of women and men; (f) Understand social norms on masculinity and the social pressures of being a man; (g) Increase awareness about the existence of power in relationships and its impact on individuals and relationships; (h) Identify different types of violence that may occur in intimate relationships and communities; (i) Explore issues and challenges faced by men who are victims of violence; (j) Discuss the relationship between the violence that men suffer and the violence they use against others; and (k) Describe Tanzanian laws and policies related to gender-based violence. Module 1 begins with an activity for participants to see how others view gender differences, roles, and inequalities. Then, they learn about gender, sex, gender equality, gender equity, and gender norms through a flipchart and group discussions. Participants will use role-play activities, case studies, discussions, presentations, and flipchart activities to explore power and violence in relationships and their impact.**
*Adaptation to Model 1:* (a) define HIV and AIDS; (b) understand the modes of HIV transmission; (c) describe how HIV is diagnosed; (d) identify HIV prevention methods; (e) describe types of HIV prevention methods; (f) describe the need for HIVST; (g) identify concerns regarding the implementation of HIVST; and (h) describe ethical considerations to enable effective implementation (see Table 1). Module 1 begins with participants learning the correct definitions for HIV and AIDS, the modes of transportation, how it is diagnosed, and some types of tests. Participants will then review HIV prevention methods, learn why HIVST is needed, the ethical considerations, and the risks and benefits of HIVST.
*Module II: Working with Men on HIV and AIDS,* (a) develop a better understanding of the reasons for, and benefits for, working with men on HIV and AIDS; (b) promote greater awareness of the links between how men are raised and the health risks they face; (c) discuss situations in the life of young men that put them at risk of STI’s, HIV/AIDS, and/or unplanned pregnancy and to identify sources of support to reduce these risks; (d) discuss the sexual transmission of STI’s and HIV/AIDS; (e) discuss the stigma that people living with HIV/AIDS face; and (f) understand the basic facts about HIV and AIDS. Module II begins with a small group discussion about why men are working with HIV and AIDS and other health risks that men may face. Participants will then complete group activities about situations in life when they put themselves at risk of STIs, HIV/AIDS, and/or unplanned pregnancy. Review of facts and case studies about the stigma faced by PLWHIV
*Module I: Making Change, Acting,* (a) identify the reasons why men might be interested in changing gender norms; (b) identify key roles that men can play in promoting health; (c) identify ways in which men can hold each other accountable in being gender-equitable; (d) discuss key concepts related to community engagement; (e) identify different types of community engagement activities; (f) help community members or groups plan a theatrical performance about gender, HIV, and male engagement; (g) understand the principles of social marketing and practice the Training Steps for developing a Community-based or mass media campaign; (h) help community members or community groups plan a health fair on gender, HIV, and male engagement; (i) develop the skills necessary to conduct group discussions around gender and HIV; (j) identify methods of using sports to reach men and the community; (k) provide participants with the skills to make door-to-door visits in their communities; and (l) discuss strategies for reaching men through peer outreach. In module III, participants are educated on ways men are interested in changing gender norms and promoting health and gender equitability through activities with small groups and discussions. Participants are then educated on community engagement activities, including a theatrical performance about gender, HIV, and male engagement, a mass media campaign, a health fair on HIV and gender, group discussions on HIV and gender, outreach through sports, door-to-door visits, and one-on-one discussions. This is achieved through small group brainstorming, discussions, and demonstrations of each community engagement activity.
*Module IV: Men Under 35*. Provide strategies for facilitators who want to host a meeting to initiate a MEN UNDER). Module IV begins with several questions reflected on, discussed in small groups, then discussed in the whole group. The groups will then create a shared vision and mission statement for the Men Under 35 group.
*Added Module V: Effective Communication and Counseling Skills*. (a) define effective communication; (b) explain factors that facilitate effective communication; (c) explain barriers to communication; (d) define counseling; (e) describe the essential skills for counseling; (f) explain the traditional HIV risk-reduction counseling, and (g) describe the recommended HIV risk reduction approach. Module V is focused on giving the participants effective communication and counseling skills. It begins with educating the participants on effective communication and basic counseling skills. SOLER, a technique for demonstrating interest and interest non-verbally, is introduced and the importance of immediacy and barriers to communication.
*Added Module VI: HIV Self-Testing*. (a) define the HIV Self-Testing concept; (b) discuss the benefits of HIV Self-Testing; (c) explain the methods of HIV self-tests to be used in the pilot; (d) describe the type of self-tests available; (e) explain the benefits of confirmatory HIV testing for self-testers and differentiate “triaging” from “confirmatory” tests; (f) explain the components of the oral HIV self-test kits; (g) demonstrate step by step how to perform HIV Self-Testing accurately using OraQuick; (h) be able to read and interpret HIV test results using OraQuick; (i) explain the benefits of linking to HTs after self-testing for both negative and positive client; (j) outlines perceived barriers for linkages to care and strategies to overcome those barriers; (k) discuss how to detect the indicators of social harm; (l) define the roles and responsibilities of the CBHW/ peer educators for the community-based distribution model; (m) describe Standard Operating Procedures (SOPs) for HIVST. In this module, participants will be educated on the definition of HIVST, the benefits, and the different types of Self Testing methods. Then, there will be an HIVST demonstration using OraQuick. This demonstration will include conducting the test and interpreting the results. Participants will then learn about the benefits of linking HIV care and treatment, social harm, and incident identification and reporting.

**Table 2:** Training Agenda for Community-based HIV Providers

Training Objectives	Topics /Activities	Projected Agenda
• To understand the difference between “sex” and “gender.” • To understand the terms “gender equity” and “gender equality.” • To understand gender issues and concerns in relation to access to HIV/AIDS care treatment and support services. • Role of men in Promoting healthseeking behavior among men and community at large • To develop a better understanding of the reasons for and benefits of working with men on HIV and AIDS.	• Looking at our attitudes (Module 1.1/ 1.3) • Learning about Gender and Sex (Module 1) • Act like a Man/Act like a Woman- • What is Power • Persons and Things • What is violence, types, and forms of violence in relation to the HIV/AIDS epidemic (Module 1.7) • Why Work with Men on HIV and AIDS (Module 2.1) • Men’s Role in Health Promotion. • I Am at Risk When (Module 2.4)	Day 1
• To promote great awareness on men and heath • To discuss the sexual transmission of STIs and HIV and AIDS • To understand the basic facts about HIV and AIDS.	• Transmission of HIV and AIDS: A Signature Hunt (Module 2.5) • HIV and AIDS Myths and Facts (Module 2.7) • Men, Gender, and health - role-plays and reflections • Activity 3.2: HIV Testing and Counselling (MODULE 2 manual) • To Understand stigma and discrimination in relation to HIV testing and linkage to treatment and care. • Activity 5.2: People Living with HIV–Antiretroviral Therapy (MODULE 2 manual) • Experience of being stigmatized and how it affects access to Health care services. • Stigma and discrimination associated with HIV/AIDS, access to care, treatment, and support services (Module 1.) • Are men interested in change? • Making change, taking action • Men and Health: Caring for Oneself: Men, Gender, and Health	Day 2
• Define HIV Self Testing concepts • Discuss the benefits of HIV Self Testing • Explain the methods of HIV Self Testing to be used in the pilot • Describe the types of HIV Self Testing available • Explain the benefits of confirmatory HIV Testing • Describe the various service delivery approaches of HIV Self Testing.	• HIV Self-Testing • Concept of HIV Self-Testing • Definition of HIV Self-Testing (HIVST) • Benefits of HIVST • HIV Self Testing method • Triaging versus Confirmatory Tests • Approaches to HIVST	Day 3
• Effective use and management of HIVST Kits • Strengthen Linkage to HIV Care and treatment services	• HIVST Demonstration • Component of HIVST Kit • Steps to conduct the test • Interpretation of Results • Cross-cutting issues of HIVST • Barriers to linkage to care and treatment • Ways of promoting linkage • Linkage Mechanisms/Tools (Directory of referral sites/hotlines/mapping referral points for different health services suitable for men) • Social harm • Incident Identification and Reporting	Day 4
• To identify key groups and how to reach them • To identify the primary message that should be conveyed in community activities on male engagement and gender • To examine the possibility, advantages, and challenges of building a new alliance • To increase the effectiveness and reach of efforts to engage men in getting 95 95 95 • To understand monitoring and evaluation – HIVST	• Understanding different strategies of reaching high-risk men • Mapping, identifying high-risk men, and mobilizing men for HTS – Hotspot analysis, contact mapping, network mapping, Gap analysis on the services available. • Action plans • Weekly/monthly Performance tracking Tools • Linkage mechanism and tools • Follow up visit forms by community volunteers/men’s champions • Directory of referral sites/hotlines/mapping referral points for different health services suitable for men.	Day 5

**Table 3:** Nomination Criteria for Community-based HIV Providers

CHBSPs Ideal Characteristics	
Demographics	18 years old and above and male
	Current member of an intervention camp
Technical Skills	Possesses public speaking skills and can convince men to attend non paid health events/forum
	Able to commit to the work program, including irregular hours, nights and weekends
	Voluntarily test for HIV regularly and influence others for HIV testing and counseling (HTC) services and have strong commitment with respect to program focus.
Interpersonal Skills	Someone peers would feel comfortable talking about HIV testing and HIV self-testing
	Someone who would encourage his peers to access HIV testing and counseling services, support enrollment to care and treatment centers and ensure adherence
	Knowledgeable about the local context and setting; especially networks of his peers and issues around them
	Tolerant and respectful of other communities where differences may exist
	Demonstrate reliability, positivity, flexibility and initiative during volunteer work in supporting other men for HTC services
	Demonstrate ability to inspire other /strong role model for the behavior they seek to promote with others and have self-confidence and leadership qualities
	Comfortable talking about sexual related issues, personal and taboo topics, or be able to develop that quality during training in relation to men access to care treatment and support services

**Table 4:** Phases of the ADAPT-ITT model for the STEP Intervention

Phases 1–8	Project Activities
Phase I: Assessment (2015–2016)	• Conducted cross-sectional survey of men (n = 878) assessing men’s willingness to self-test • 67% of men who had never tested (n = 301) were willing to self-test, and 72% of men who had previously tested (n = 577) were willing to self-test • In-depth interviews with 23 men found demand-creation strategies included formal and informal conversations, and peer accompaniment to clinic • Privacy, stigma reduction, and being the first to know their HIV test result were all mentioned as benefits of HIVST
Phase II: Decision (2018)	• Lack of policy regarding HIVST and availability of HIVST kits delayed this phase • Research team met with Director of National AIDS Control Programme (NACP), HIVST stakeholders, and program officers from Sauti project to provide expertise on training community-based health service providers (CBHSP) for demand generation • Used information from Assessment Phase and stakeholder input to decide to recruit CBHSPs and train them with an adaption of the Sauti Project’s “National Training for HIV Prevention using HIV Self Testing” manual
Phase III: Adaptation (2018)	• Used manual to adapt training to CBHSPs for implementation of STEP intervention • Topics included HIV prevention methods, HIVST risks and benefits/ethics, other men’s health issues (STI’s, unplanned pregnancy), challenge gender norms, effective communication/counseling, use of HIVST, and benefits of HIV care initiation
Phase IV: Production (2018)	• The final manual consisted of six training modules including: 1) Introductory Activities; 2) Working with Men on HIV and AIDS; 3) Making Change, Acting; 4) Men Under 35; 5) Effective Communication and Counseling Skills; 6) HIV Self-Testing (outlined in Table 2) • Manual outlined methods of training to include: theater/roleplaying, PowerPoint lectures, question and answer, group presentations, and brainstorming with a pre- and post-test to evaluate learning • Training would enable CBHSPs to create demand for community-based HIVST distribution, led by a nurse at a tent-site near the camp from which participants were recruited
Phases V and VI: Topical Experts and Integration (2018)	• Team of experts and community members including a monitoring and evaluation specialist, local government authorities, community/project leaders, reviewed STEP training manual and provided feedback during one-day technical meeting • Theater-tested intervention and received feedback from experts to consider recruiting CBHSPs from local networks of men known as “camps” • Experts also recommended screening for suicidal ideation of participants prior to enrollment in study and receipt of HIVST
Phases VII and VIII: Training and Testing (2019)	• Research team met with 166 members of 9 “camps” in target area to discuss nomination of CBHSPs in accordance with recruiting practices and qualifications of CBHSPs utilized by Sauti project officers • “Camp” members were instructed to nominate 2–3 members of their camp to be CBHSPs, which led to the nomination of 26 men across 9 camps • 25 men attended 5-day training session which included 6-module curriculum and opportunities for self-reflection, as well as discussion regarding stigma reduction and promotion of HIVST to camp members • 25 CBHPs had approximately one month to educate peers within their camps and recruit for participation in the STEP study using communication and social influence strategies • Recruited participants who reported to the tent-site provided informed consent, took a baseline survey, and received HIVST education from a healthcare worker on how to use the HIVST kit, interpret results, and where to receive follow-up care

**Table 5:** Baseline survey characteristics for men in intervention group

Characteristics	Frequency	Percentage (%)
Age		
18–22	62	26.3
23–27	66	28.0
28–32	42	17.8
33+	66	28.0
Education level		
Primary school or less	123	52.1
Some secondary school	36	15.3
Completed Secondary or more	77	32.6
Marital status		
Ever married	94	39.8
Never married	142	60.2
Role in camp		
Camp leader	23	9.8
Camp member	213	90.3
Frequency of visiting camp		
Daily	146	61.9
Weekly	72	30.5
Monthly	18	7.6
Employment status		
Does not have paid job	102	43.2
Has paid job	134	56.8
Have you ever paid for sex?		
One or more times a month	62	26.3
Never paid for sex	174	73.7
Has sexual partners		
Yes	198	83.9
No	38	16.1
Consistent condom use		
Always	43	18.2
Sometimes	67	28.4
Never	124	52.5
Missing	2	0.9
Do you know partner’s HIV status?		
Yes	110	46.6
No	88	37.3
Missing	38	16.1
Ever heard about HIVST		
Yes	127	53.8
No	109	46.2
Who encouraged you to take an HIV self-test?		
Family member	26	11.0
Health worker	10	4.2
Peer supporter	200	84.8
Talked to sexual partner about HIVST		
Yes	69	29.2
No	129	54.7
Missing	38	16.1

**Table 6:** Self-reported HIV self-testing behavior of men in intervention group at 1-month follow-up (n = 236)

Characteristics	Frequency	Percentage (%)
Were you provided with HIVST?		
Yes	236	100.0
No	0	0.0
Where were you when you used the self-test?		
At the tent	12	5.1
At a private space far from the tent	220	93.2
Did not use it	4	1.7
What was your result?		
Negative	218	92.4
Positive	10	4.2
Invalid	4	1.7
Missing	4	1.7
What did you do after self-testing?		
Went to health facility	7	3.0
Visited the study tent	95	40.3
I did nothing	125	53.0
Missing	9	3.8
How many days after you were provided with the HIVST did you use it?		
Same day	160	67.8
After the first day	72	30.5
Missing	4	1.7

**Table 7:** Perceptions of HIV self-testing among participants who used the kits

Characteristics	Frequency	Percentage (%)
How easy was it to understand the HIVST kit instructions?		
Very easy	42	17.8
Easy	64	27.1
Normal	107	45.3
Difficult	4	1.7
Missing	19	8.1
What do you like about the HIV self-test kit?		
No need for needle prick	12	5.1
It has more confidentiality	220	93.2
It is quick and easy to use	4	1.7
In general, how satisfied are you with the HIV self-test kit are?		
Normal	9	3.8
Satisfied	83	35.2
Very Satisfied	124	52.5
Not Satisfied	1	0.4
Missing	19	8.1
Which HIV testing method would you like to use in the future?		
HIVST	207	87.7
Convention HIV test	29	12.3
If it happens that you are required to pay for HIV, self-test kit, would you be willing?		
Yes	218	92.4
No	18	7.6

**Table 8:** Mobile health and HIV self-testing

Characteristics	Frequency	Percentage (%)
Do you have a mobile phone?		
No	11	4.7
Yes	225	95.3
Did you use your phone to take pictures of HIV test kit results?		
No	216	91.5
Yes	9	3.8
Missing	11	4.7
If yes, did you show anyone your HIV test results?		
No	2	22.2
Yes	7	77.8
Did you call anyone and share the results of your HIV self-test?		
No	170	72.0
Yes	47	19.9
Missing	19	8.1
Did you send a text message to anyone about the results of your HIV self-test?		
No	209	88.6
Yes	27	11.4

## Data Availability

Given the sensitive nature of the personal data collected during this study, the data is not publicly available. The data is currently stored in a secure cloud-based storage platform that can be accessed by the Principal Investigator. Requests for de-identified data can be made available on request.

## Abbreviations

ADAPT-ITT: Assessment, Decision, Adaptation, Production, Topical Experts, Integration, Training, Testing
AGYW: Adolescent Girls and Young Women
ART: Antiretroviral Therapy
CBHSP: Community-Based Health Service Providers
FSW: Female Sex Workers
GoT: Government of Tanzania
HIVST: HIV Self-Testing
HTS: HIV Testing Services
KVP: Key and Vulnerable Population
MLWH: Men Living with HIV
MSM: Men who who sex with men
NIMART: Nurse-initiated management of ART
NACP: National AIDS Control Program
PEPFAR: President’s Emergency Plan for AIDS Relief
PLWH: People living with HIV
PrEP: Pre-exposure prophylaxis
STEP: Self-Testing Education and Promotion
TACAIDS: Tanzania Commission for AIDs
USAID: United States Agency for International Development
